# Inhibition of apoptosis signal-regulating kinase 1 alters the wound epidermis and enhances auricular cartilage regeneration

**DOI:** 10.1371/journal.pone.0185803

**Published:** 2017-10-18

**Authors:** Qian-Shi Zhang, Deepa S. Kurpad, My G. Mahoney, Marla J. Steinbeck, Theresa A. Freeman

**Affiliations:** 1 Department of Orthopaedic Surgery, Sidney Kimmel Medical College, Thomas Jefferson University, Philadelphia, PA, United States of America; 2 Department of Spine Surgery, The Second Xiangya Hospital of Central South University, Changsha, Hunan, P.R. China; 3 Department of Dermatology and Cutaneous Biology, Sidney Kimmel Medical College, Thomas Jefferson University, Philadelphia, PA, United States of America; 4 School of Biomedical Engineering, Science & Health Systems, Drexel University, Philadelphia, PA, United States of America; New York Medical College, UNITED STATES

## Abstract

Why regeneration does not occur in mammals remains elusive. In lower vertebrates, epimorphic regeneration of the limb is directed by the wound epidermis, which controls blastema formation to promote regrowth of the appendage. Herein, we report that knockout (KO) or inhibition of Apoptosis Signal-regulated Kinase-1 (ASK1), also known as mitogen-activated protein kinase kinase kinase 5 (MAP3K5), after full thickness ear punch in mice prolongs keratinocyte activation within the wound epidermis and promotes regeneration of auricular cartilage. Histological analysis showed the ASK1 KO ears displayed enhanced protein markers associated with blastema formation, hole closure and regeneration of auricular cartilage. At seven days after punch, the wound epidermis morphology was markedly different in the KO, showing a thickened stratum corneum with rounded cell morphology and a reduction of both the granular cell layer and decreased expression of filament aggregating protein. In addition, cytokeratin 6 was expressed in the stratum spinosum and granulosum. Topical application of inhibitors of ASK1 (NQDI-1), the upstream ASK1 activator, calcium activated mitogen kinase 2 (KN93), or the downstream target, c-Jun N-terminal kinase (SP600125) also resulted in enhanced regeneration; whereas inhibition of the other downstream target, the p38 α/β isoforms, (SB203580) had no effect. The results of this investigation indicate ASK1 inhibition prolongs keratinocyte and blastemal cell activation leading to ear regeneration.

## Introduction

Mammalian epimorphic regeneration is limited to ear tissues and digit tips in a few strains of laboratory mice. These strains include the Murphy Roths Large (MRL) mouse, the p21 knockout (KO)) mouse and the doxycyclineinducible Lin28a transgenic mouse [1–3]. In contrast, limb amputation in lower vertebrates generates a specialized wound epidermis, a tissue reminiscent of the apical ectodermal ridge (AER) in limb development, created to direct blastemal formation, and through epimorphic regeneration this region recreates the lost appendage. Classical experiments have shown transplantation of the wound epidermis (with innervation) or the AER, is sufficient to drive the formation of an entirely new limb [4, 5]. It is widely believed the regenerative ability of the wound epidermis has been lost as mammals evolved; and the need for fast, efficient wound healing and functional restoration of the epithelial barrier superseded the need to regenerate missing tissues. Interestingly, the wound epidermis of the regenerating MRL mouse differentially express genes characteristic of an early regenerative keratinocyte activated-like state, which may contribute to their regenerative abilities [6].

Based on extensive studies in lower vertebrates, it is believed the wound epidermis promotes formation of the blastema that directs cells to undergo “de-differentiation”, proliferation and re-differentiation. However, a detailed analysis of these events has not been performed in mammals and a complete understanding of how the blastema controls cartilage regeneration remains elusive. Studies of the blastemal region of mice capable of regeneration have revealed differences in cellular activities including; increases in energy metabolism, DNA damage, G2/M cell cycle arrest, metalloproteinase activity and cellular proliferation[7, 8]. A very recent study showed that stabilization of HIF-1α over 10 days after ear punch induces regeneration, and concludes that HIF-1α may be a master regulator of the above events ^[^^9^^]^.

Recently, during our investigation of the mitogen-activated protein kinase kinase kinase 5 (MAP3K5), Apoptosis Signal-regulated Kinase-1 (ASK1) involvement in cartilage and bone formation [10, 11], we found the ASK1 KO mouse ear holes also closed after being punched. ASK1 is involved in a wide range of cellular processes including stress-related responses, cytokine and growth factor signaling, cell cycle control, differentiation and apoptotic cell death [12, 13]. Several studies have shown knockout or inhibition of ASK1 affects these processes, thereby reducing cell death, the inflammatory response and tissue damage after injury [10–12, 14]. Based on previous studies showing ASK1 promotes both keratinocyte and chondrocyte terminal differentiation [10, 15], the regenerative ability of the ASK1 KO mouse was investigated to determine if the ASK1 effect on keratinocyte differentiation in the wound epidermis would enhance regeneration, and how ASK1 inhibition would affect blastemal formation and cartilage regeneration.

In this study, we show inhibition of ASK1 results in prolonged activation of keratinocytes in the wound epidermis, slower restoration of the epithelial barrier and enhanced auricular cartilage regeneration. At day 7 after punch, cellular activities in the blastemal area of the ASK1 KO mouse included; increased metalloproteinase production, DNA repair, mesenchymal cell proliferation and a decrease in p21 expression, similar to those observed in other regenerative mice. Regeneration was also enhanced when inhibitors of ASK1, or the upstream activator (CAMKII), or downstream effector (JNK) were topically applied to the skin of the wounded ear throughout the healing process. Taken together, these findings support a role for ASK1 as an inhibitor of mammalian regeneration, as it promotes keratinocyte differentiation and closure of the epithelial barrier, which may limit blastemal formation and retard auricular cartilage regeneration.

## Results

### Loss of ASK1 promotes ear regeneration in C57BL6 mice

**Fig 1:**
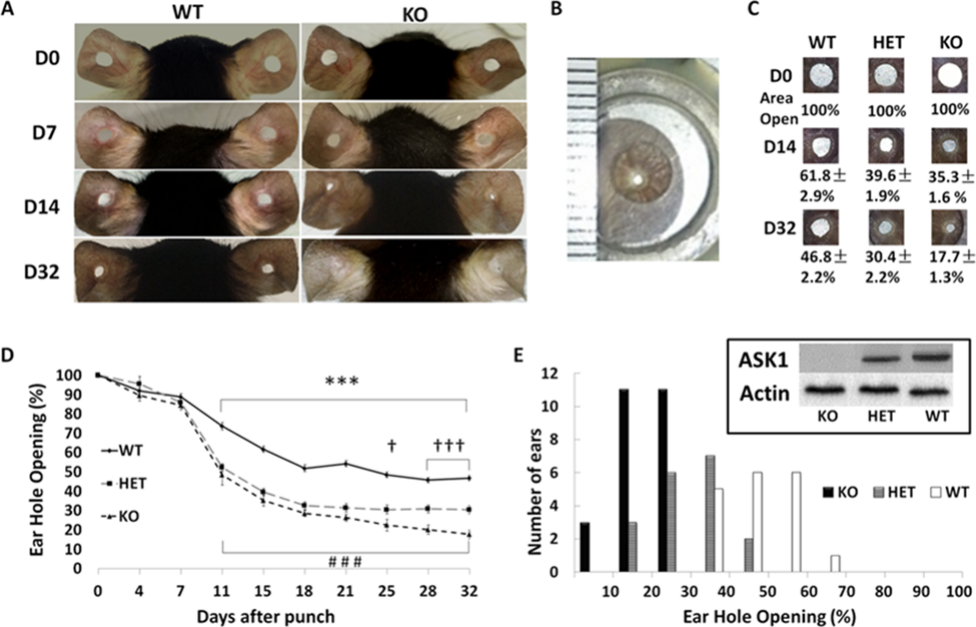
Loss of ASK1 increases ear hole closure. A) Two-millimeter holes were punched in ears on day 0 and closure was photographed at days 0, 7, 14 and 32 for WT and KO mice. B) A magnetic ear clip flattened the ear for photographing. C) Representative images of ear closure and average percent of areas closure are shown on D14 and D32 measured for WT, HET, and KO. Data are presented as mean ± SEM, ASK1 KO mouse ears showed accelerated, and greater amounts of wound closure compared to HET and WT ears. D) ASK1 KO mouse ears had a closure rate of 64.7± 1.6% by area on D14 after punch, which was more efficient than HET and WT ears which closed 60.4± 1.9% and 38.2±2.9%. On the D32 after punch ASK1 KO mouse ear hole closed 82.3±1.3% when HET and WT ears closed 69.6±2.2% and 53.1±2.2%. E) The analysis of frequencies of ear punch closure on the D32 after punch in WT, HET and KO mice. Inset shows Western blot with protein levels for ASK1 and actin for each genotype. p < 0.05 (one asterisk/mark), p < 0.01 (two asterisks/mark), p < 0.001 (three asterisks/mark). KO Vs. WT (*); KO Vs. HET (†); HET Vs. WT(#).

To evaluate wound healing and tissue regeneration a 2-mm hole was made in the ear of 8-week-old C57BL6 wild type (WT), ASK1 heterozygous (HET) and ASK1 knockout (KO) mice. Fig 1A shows representative images ear hole closure in WT and KO mice at days 0 (D0), 7 (D7), 14 (D14) and 32 (D32) after punch. The ear hole is flattened by placement in a magnetic clip (Fig 1B) to assure images represent accurate hole sizes as shown in Fig 1C at D0, D14 and D32; with the average percent opening calculated as a percentage of the D0 ear hole opening. The ASK1 KO mouse ears showed accelerated, extensive hole closure compared to HET and WT ears (Fig 1C). The average percent opening on D14 by area was 35.3±1.6% (KO, n = 25) compared to 39.6±1.9% (HET, n = 18) and 61.8±2.9% (WT, n = 18). On D32, the KO opening was only 17.7±1.3% compared to 30.4±2.2% for the HET and 46.8±2.2% for WT mice. The complete timeline of ear closure at each day indicates that from D11 to D32 the KO and HET ear hole openings were significantly reduced compared to WT (Fig 1D). Compared to the HET, the KO ears had enhanced regeneration from D21 to D32, (p < 0.05 (one asterisk/mark), p < 0.01 (two asterisks/mark), p < 0.001 (three asterisks/mark). KO vs. WT (*); KO vs. HET (†); HET vs. WT (#)). The analysis of frequencies of ear punch closure on D32 after punch is shown in Fig 1E (WT mice, white box; HET, gray box; and KO, black box). The Fig 1E inset, shows a Western blot of ASK1 protein from each animal based on genotype, and indicates the KO has complete loss of ASK1 protein and the HET has half the ASK1 protein, as expected. Thus, enhanced ear hole closure is directly correlated with ASK1 protein level.

### Auricular cartilage regeneration is enhanced in ASK1 KO mice

**Fig 2:**
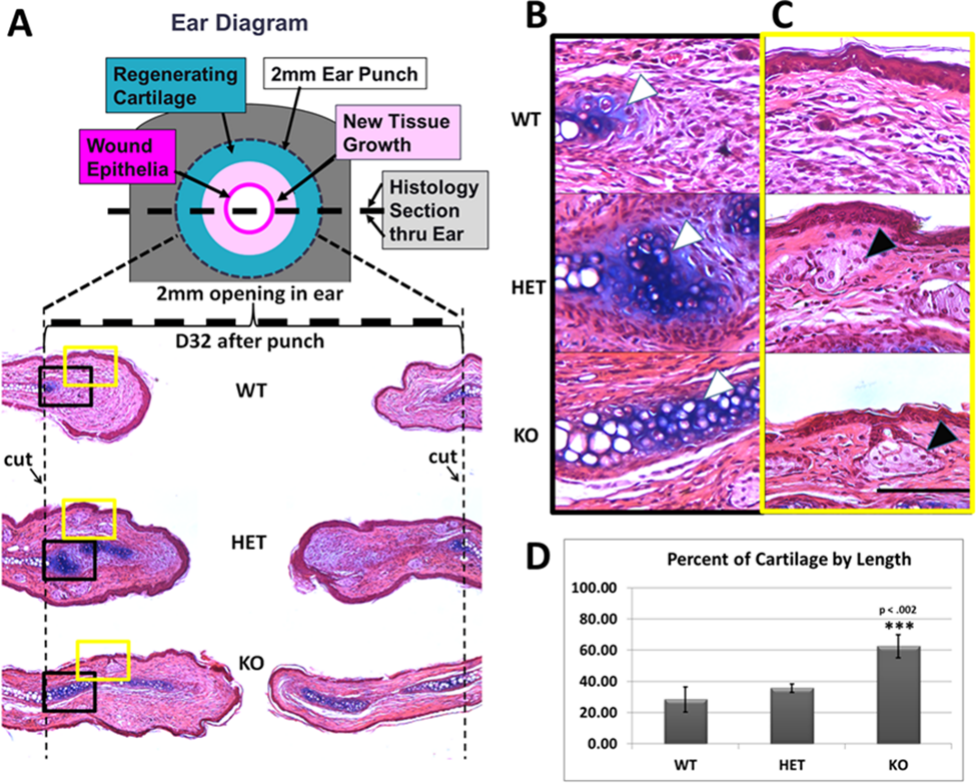
Regeneration of cartilage and ear tissues. A) A diagram of the mouse ear shows placement of the ear punch and the localization of the regenerating tissues. The thick dashed line indicates where the tissue section below was obtained and the dotted line demarcates the original and newly regenerated tissue in the hematoxylin and eosin (H&E) and alcian blue staining of WT, HET and ASK1 KO ears on D32 after punch. In the WT ear very little new cartilage (darker blue) has regenerated as compared to the HET and ASK1 KO ear. B) A higher magnification image of the black box in A showing regenerated cartilage (white arrow) C) A higher magnification image of the yellow box in A highlighting the presence of sebaceous glands (HET and KO–black arrow) Scale bar = 500 μm.

Histological sections collected at D32 after punch (thick dashed line in diagram Fig 2A) showed a greater amount of new tissue formation within the punched area for the KO mouse (Fig 2A, representative sample of n = 3 mice/ genotype). The new tissue growth in the KO nearly spanned the 2mm space (area between black dotted lines) whereas, less new tissue was observed in the HET and less in the WT ears. The regenerated cartilage (dark alcian blue stained tissue) within the newly developed tissue of the ASK1 KO mouse was extensive and formed a straight line approaching the epitdermis (dark pink). In comparison, there was less regenerated cartilage in the WT and HET ears. The black box (Fig 2A) depicts a region of regenerated cartilage at higher magnification (Fig 2B; white arrowhead). The regeneration of other ear structures, including sebaceous glands, (Fig 2A; yellow box; Fig 2C higher magnification) were present along the epidermis of the HET and KO mice (Fig 2C; black arrowhead). The percentage of the length of cartilage regeneration in the newly formed tissue was 62.5% for the ASK1 KO, 35.7% in the HET ears, and significantly less, 28.4% in the WT (p ≤ 0.002; Fig 2D). It is important to note closure of the ear is not synonymous with cartilage regeneration, as the ear hole can be 100% closed but have no cartilage regeneration.

### Delayed reformation of basement membrane and mature epidermis in ASK1 KO mice

**Fig 3:**
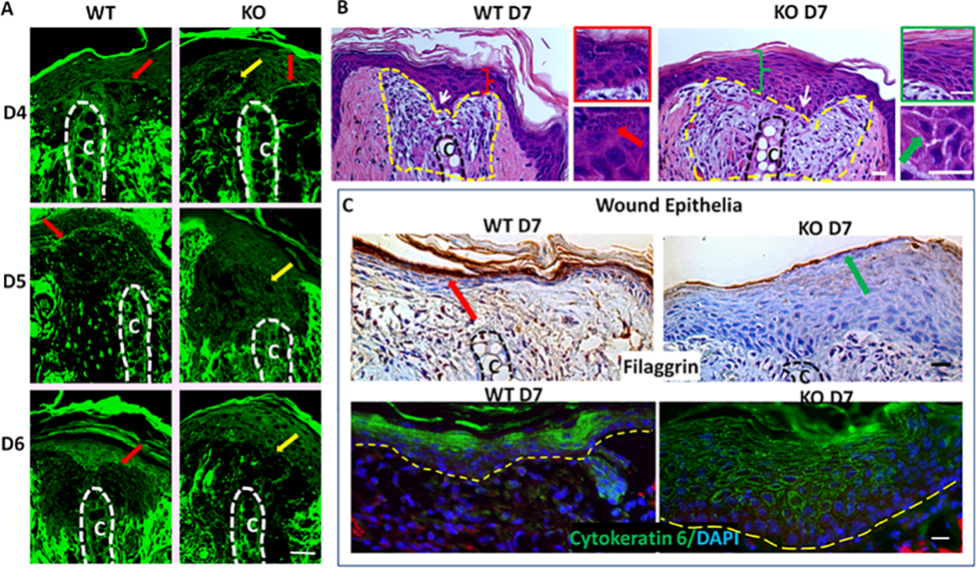
The ASK1 KO mouse has delayed basement membrane formation and maturation of the wound epidermis. A) Basement membrane forms between the epidermis and dermis and is observed in the WT at D5 after punch (red arrowheads) but is absent in the ASK1 KO ear (yellow arrowheads, cartilage indicated by dotted line and “C”) B) At D7 the healed epidermis of the WT mice was compact (red bracket; high mag in red box) and basal keratinocytes (white arrows) are mostly flattened. The KO mice epidermis was thickened (green bracket; high mag in green box) with enlarged columnar basal cells (white arrows). The granular layer and keratohyalin granules are observed in the WT (red arrow) not in the KO. In the KO, the epitheliun showed extensive parakeratosis and a compact stratum corneum (green box). Additionally, intercellular spaces of the spinous layer were widened (green arrow). C) Staining (dark brown) for the terminal differentiation marker, filaggrin is increased in WT. In the KO cytokeratin 6 (green, DAPI–blue) is widely expressed in cells of the spinosum and granulosum layers of the epidermis. Scale bars = 100 μm.

Autofluorescence of eosin in H&E sections was used to visualize the extracellular matrix and basement membrane formation, as previously reported for MRL versus C57BL6 ear regeneration [16]. In the ASK1 KO wound epidermis, the basement membrane (Fig 3A) was partially absent at D4 and absent in D5 and D6 (yellow arrows). Whereas, the basement membrane was always present in the WT wound epidermis (red arrows). The existing cartilage is marked with a dotted line and the letter “C”.

Further investigation of the wound epidermis showed that while ASK1 was not required for re-epithelialization of the wound, terminal differentiation of the keratinocytes in the ASK1 KO mouse was delayed (Fig 3B). There were several abnormal features that support this observation. First, the wound epidermis of the WT mice was more compact (red bracket, higher mag. In red box) while the ASK1 KO mice epidermis appeared thickened (green bracket, higher mag. green box) with enlarged columnar basal cells (white arrows). The re-epithelialized epidermis in the WT also had a clear granular layer and keratin hyalin granules (red arrow) which the KO mouse lacked, suggesting delayed terminal differentiation of keratinocytes (comparing red vs. green box). Second, an extensive parakeratosis and a compact stratum corneum were also observed in the KO mouse (green box). Furthermore, the presence of wider intercellular spaces between cells of spinous layer suggested abnormal adhesion of keratinocytes (green arrow). These observations were consistent for the majority of the histological images (n = at least 15 ears from each genotype). Taken together, these findings indicate the KO mouse has a less mature wound epidermis and a basement membrane that was slower to reform, a requirement for blastemal development. In support of this, note the enlarged blastemal region (inside yellow dashed line) below the wound epidermis in the KO, as compared to that of the WT. This region is composed of loose connective tissue with a lesser amount of extracellular matrix, as opposed to the dense connective tissue regions on either side (darker pink).

### Wound epithelial keratinocytes exhibit delayed terminal differentiation in ASK1 KO mice

To further investigate the keratinocyte differentiation status, D7 wound epidermis was immunostained for the terminal differentiation marker filament aggregating protein (filaggrin) (Fig 3C). Filaggrin was localized to the statum corneum of the WT mice (red arrow). In contrast, a significantly reduced amount of filaggrin was observed in the ASK1 KO (green arrow), indicative of a delay in keratinocyte terminal differentiation (n = 3 each genotype). No differences were observed between KO and WT uninjured skin immunostained with filaggrin (S1 Fig). Immunofluorescent localization of cytokeratin 6, a protein expressed in activated keratinocytes was expressed in both WT and ASK1 KO epithelia (above yellow dotted line; Fig 3C) at D7. However, WT expression of cytokeratin 6 was confined to the dead and dying cells of the stratum corneum whereas; in the ASK1 KO each individual cell within the stratum spinosum and granulosum contained cytoplasmic cytokeratin 6 (n = 3 each genotype).

### Loss of ASK1 promotes cellular behavior characteristic of blastemal formation

**Fig 4:**
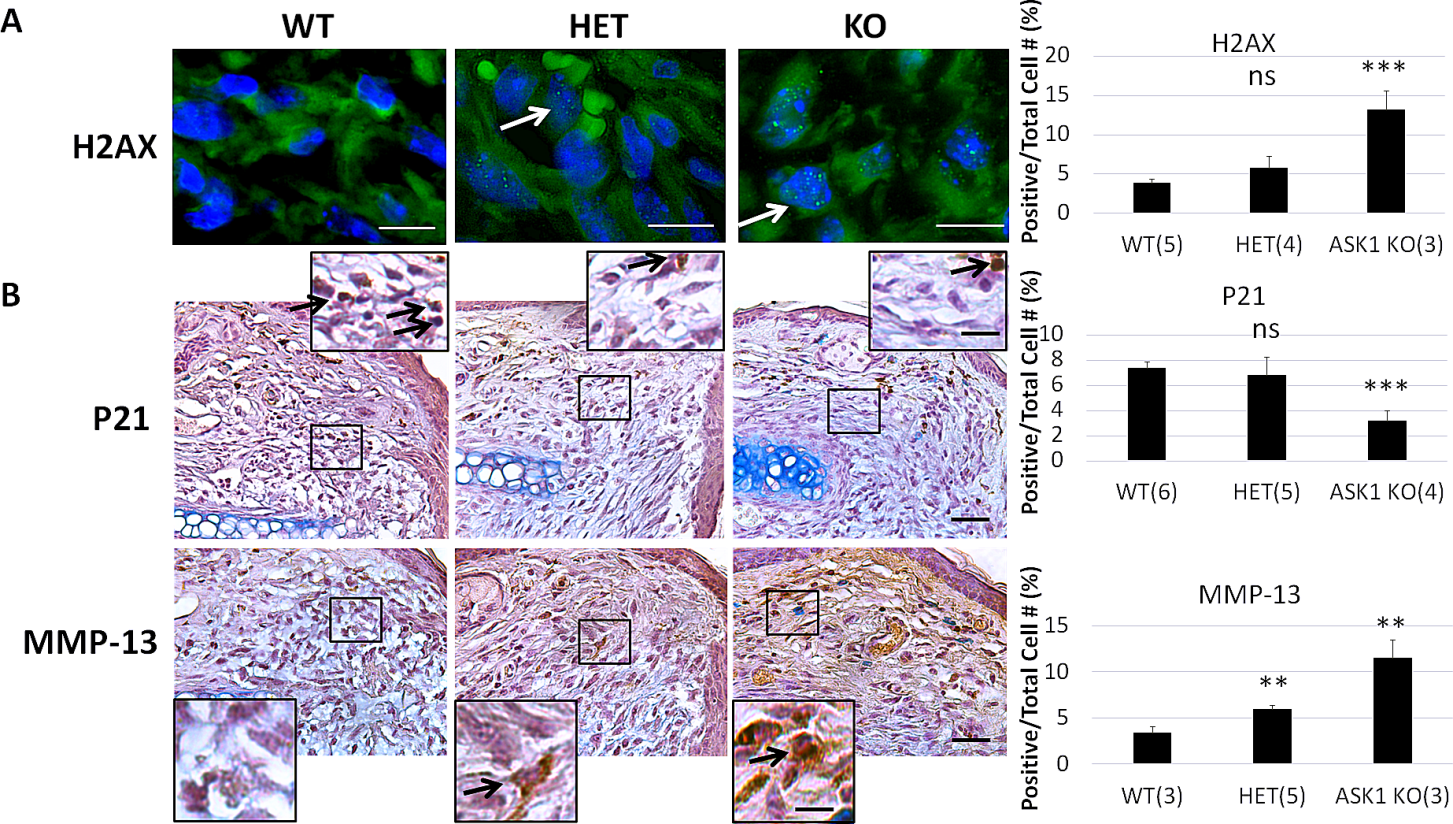
Protein markers indicating cellular activities of blastema formation were more prevalent in the ASK1 KO. A) Sections were stained for H2AX immunofluorescence, positive green nuclear fluorescent dots (white arrows) were consistently observed in the ASK1 KO tissue. The percentage of H2AX-positive cells was determined by counting all the positive cells per total cells in blastemal area in 3 fields from 3 mice. Data are presented as mean ± SEM. B) Similar DAB immunohistochemistry was performed with antibodies to p21 and MMP-13 positively stained cells are shown at black arrows in high magnification inset. Percent of positive stained cells/ total cells was determined and graphed for WT, HET, and ASK1 KO mice. White scale bar = 100 μm, black scale bar = 500 μm in low mag image and 100 μm in high mag. inset.

Blastemal formation is differentiated from normal wound healing by the presence, localization and expression levels of several key proteins. To determine how the loss of ASK1 affects these proteins, we performed immunohistochemistry on D7 post-punch ear tissues for all three genotypes. Immunofluorescent staining showed the DNA repair protein H2AX is present in nuclei of cells within the blastema of ASK1 KO ears (white arrow; Fig 4A, white bar = 100μm). Quantification showed 13% of nuclei per total cell number were positive in the ASK1 KO (p < 0.001), 6% in HET and 4% in WT tissue. Representative images of DAB immunochemistry staining for p21 and MMP-13 are shown for each of the genotypes (red arrows indicate representative areas with increased positive staining; mag bar = 500μm in low mag, and 100 μm in higher mag. inset; Fig 4B). Quantification of the staining for the cell cycle protein, p21 showed a significant decrease in the KO; 3% compared to 7% for both the HET and WT (p < 0.001). The reverse trend was observed for the protease, MMP-13 (p < 0.01), which showed a statistically significant increase for the ASK1 KO mice compared to WT. MMP-13 (p < 0.01) was also significantly increased for HET mice compared to WT (black arrows on high mag insets show positive staining).

### ASK1 inhibitor NQDI-1 treatment of WT mouse ears partially recapitulates the ASK1 KO ear response

**Fig 5:**
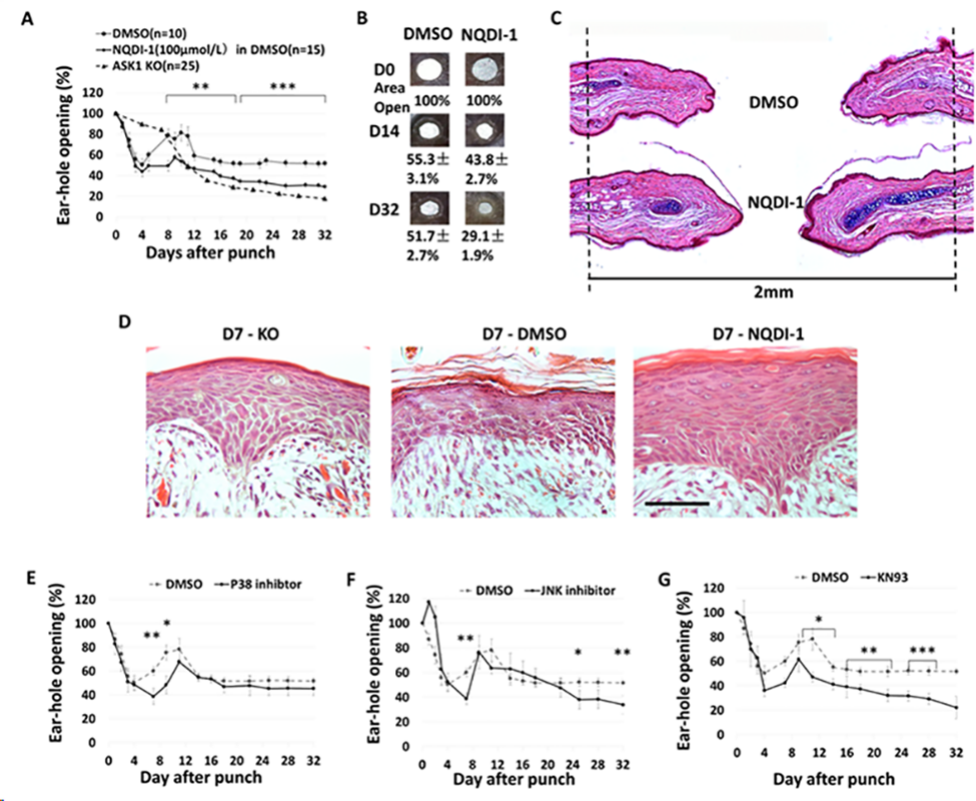
Inhibition of ASK1 or upstream activators of ASK1 enhance ear hole closure. A) Two-millimeter holes were punched in WT ears on day 0 and NQDI-1 (100μmol/L) in DMSO was added in the site of the ear hole every weekday, DMSO was added as control. Comparison of ear closure in WT mice with NQDI-1 (100μmol/L) in DMSO to WT mice with DMSO shows greater hole closure by area from day 0 to 32. B) Representative images of ear closure on D32 measured are shown for the two treatments. Average percent of areas closure are shown. Data are presented as mean ± SEM, NQDI-1 ears showed accelerated and greater amounts of wound closure compared to DMSO ear. NQDI-1 ear had a closure rate of 56.2± 2.7% by area on D14 after punch, which was more efficient than DMSO ear which closed 44.7± 3.1%. On the D32 after punch NQDI-1 ear hole closed 70.9±1.9% when DMSO ear closed 48.3±2.7%. C) Hematoxylin and eosin (H&E) and alcian blue staining of NQDI-1 and DMSO ears on D32 after punch is shown. DMSO ear shows very little new cartilage, sebaceous glands and a lot of connective tissues while NQDI-1 ear showed more new cartilage, hair follicles and sebaceous glands. D) Histology of wound epithelia shows NQDI-1 treatment slows terminal differentiation of epithelial keratinocytes at D7 after wounding, similarly to that observed in the KO. Black scale bar = 500 μm. E—G) Results of the addition of inhibitors for p38, JNK, and CAMKII on ear hole closure. t-test was used to calculate p values. Statistical significance is displayed as p < 0.05 (*), p < 0.01 (**), p < 0.001 (***).

To determine if local inhibition of ASK1 would also enhance regeneration, direct application of the ASK1 inhibitor, NQDI-1 (100μmol/L) in DMSO was topically applied before the ear was punched. The topical application at the site of the ear punch was continued for 5 consecutive days followed by 2 days of no treatment, and this regime was continued until D32. The ear hole area was measured every 3–4 days during this time and the results are expressed as percent ear hole opening based on the area of each hole on D0 (Fig 5A). During the initial 4 days the percent ear hole opening rapidly decreased, but by D8 the percentage increased indicating the initial inflammation due to swelling had resolved. This observed ear hole closing due to early swelling was more pronounced in the DMSO control as compared to the NQDI-1 treated or the ASK1 KO ears. On or around D8, the NQDI-1 treated ear was comparable to the ASK1 KO and holes in both of these ears were significantly more closed than the DMSO control ear. The ASK1 KO and NQDI-1 ears showed no significant difference to each other and gradually closed to a 18% and 29% opening, respectively at D32. At D32, the DMSO treated control ear was 52% open (n = 10, DMSO; 15, NQDI-1; 25, ASK1 KO).

Representative gross images of ear closure on D0, D14 and D32 and the average percentage of the ear hole remaining open (based on area) are shown for DMSO control and NQDI-1 treated ears (Fig 5B). The NQDI-1 treated ears showed accelerated (D14–43.8± 2.7% vs 55.3 ± 3.1%) and greater amounts of wound closure (D32–29.1± 1.9% vs 51.7 ± 2.7%) compared to the DMSO control. Histological sections from D32 (Fig 5C) showed more tissue had regrown in the NQDI-1 treated ear (distance between black dotted lines is 2mm), and a greater degree of regenerated new cartilage (dark blue) had been deposited. In Fig 5D, a comparison of the D7wound epidermis from each mouse is shown. Morphological features (thickness, cell size, stratification) of the epidermis from the NQDI-1 treated ear show similarities to those observed in the ASK1 KO (Fig 3B), and are strikingly different from the DMSO control epidermis where mature (anuclear) terminally differentiated keratinocytes are observed.

### Ear closure is also enhanced by inhibition of the ASK1 downstream target JNK, but not the α, β isoforms of p38

To determine whether local inhibition of downstream targets of ASK1 would yield similar results, the direct application of either the p38 α, β isoform inhibitor, SB203580 (100 μM) or JNK inhibitor, SP600125 (100 μM) was topically applied using the same treatment regime and measurements as the ASK1 inhibitor, NQDI-1. In response to both inhibitors a rapid decrease in the earhole was noted, in a manner similar to that observed by the DMSO control, attributed to swelling. After the initial 8 days of decreasing ear hole percent opening, a rebound to a greater percent opening was observed. The ear treated with the p38 inhibitor maintained this % opening until D32 (similar to the DMSO control; Fig 5E), while the ear treated with the JNK inhibitor gradually significantly reduced the percent ear hole opening to 34% by D32.(p < 0.01; Fig 5F).

### Inhibition of the ASK1 upstream activator, CAMKII also promotes ear closure

To determine the effects of local inhibition of an upstream activator of ASK1, the CAMKII inhibitor, KN93 was applied, and the same inhibitor treatment and measurements were followed. The early ear hole closure and reopening at D8 was observed (Fig 5G), but by D32 the treated ears showed a significant reduction in ear hole opening compared to DMSO control. Application of KN98 significantly reduced the percent ear hole opening at D10-D32 (D10-D16, 47%–39%, (p < 0.05), at D18-D26 37%–32% (p < 0.01) and D28-D32 29–22% (p < 0.001); more than that observed by the downstream inhibitors of ASK1 (n = 10 each inhibitor/DMSO treatment).

## Discussion

In this study, we show knockout of ASK1 confers regenerative ability after mouse ear punch. Hole openings were significantly reduced and greater cartilage regeneration was observed in the ASK1 KO and HET compared to WT mice. Cartilage regeneration in the KO was associated with delays in both basement membrane formation and differentiation of the wound epidermis/keratinocyte. Furthermore, increased expression of markers associated with blastemal formation was only observed in the KO mice. Topical treatment with the ASK1 inhibitor, NQDI-1, CAMKII inhibitor, KN93 or JNK inhibitor, SP600125 also enhanced tissue regeneration, whereas treatment with an inhibitor of the α, β isoforms of p38, (SB203580), had no effect. Taken together, we conclude that inhibition of the ASK1 signaling pathway alters the activation state of keratinocytes in the wound epidermis which promotes blastemal formation to promote mammalian epimorphic cartilage regeneration. Based on this and an understanding of regeneration in lower vertebrates, we propose restoring the regenerative potential of the wound epidermis by inhibiting this pathway is a critical requirement for mammalian regeneration.

Decades of research have identified a number of proteins are required for keratinocyte function during wound healing [17]. The expression patterns of these proteins may also influence regenerative competence of the wound epidermis. To date three mice have been characterized as regenerative; the MRL mouse, the p21 KO, and the *Lin-28* transgenic mouse [15, 18–21]. For each of these models, the involvement of keratinocytes in tissue regeneration is suggested; as MRL keratinocytes maintained an “activated” cytokeratin profile, p21 KO keratinocyte terminal differentiation was decreased and the Lin -28 mice had enhanced stem cell activity and hair formation [3, 6, 15, 22, 23]. A previous study has also shown that ASK1 is required for terminal keratinocyte differentiation [15]. While the focus of these studies was to identify proteins involved in mouse ear hole and appendage regeneration, the specific function of keratinocytes within the wound epidermis, as a determiner of regenerative competence was not specifically investigated. Based on the current study, it is not implausible that altered keratinocyte function in all these mouse models may control regenerative competence.

The involvement of ASK1 and both upstream CAMKII and downstream JNK inhibitors of ASK1 also promoted ear hole closure. Each of these kinases has been shown to play a role in terminal keratinocyte differentiation. Inhibition of CAMKII may cause delayed keratinocyte differentiation through deregulation of calcium signaling which is a critical regulator of ASK1 activation as well as the permeability of the epithelial barrier and the formation of tight junctions [24]. Tight junction formation directly increases with intracellular calcium levels and stimulates protein kinase C activation, which controls formation of the epidermal granular layer[25] and the expression of the terminal keratinocyte differentiation markers; profilaggrin, involucrin and transglutaminase [26–28]. Downstream, activation of ASK1 leads to the selective phosphorylation of JNK and/or p38 MAPK isoforms to initiate signaling cascades that control cytokine and growth factor signaling, cell cycle regulation, cellular differentiation, survival, hypertrophy and apoptosis [2,3]. However, only JNK inhibition and not the inhibition of the α, β isoforms of p38 enhanced regeneration; even though both have been implicated in the terminal differentiation of keratinocytes [29, 30]. This may be due to the specificity of the SB203580 inhibitor which inhibits only p38 α and β, not gamma or delta. Delta has been implicated as the most important p38 isoform, regulating keratinocyte migration, proliferation and epidermal growth factor receptor internalization [31, 32]. Unfortunately, inhibitors specific for these isoforms are unavailable and pan p38 inhibitors lack specificity.

In our previous study, we reported that loss of ASK1 results in increased cartilage production and enhanced bone formation in an ectopic endochondral ossification model [10]. A possible mechanism involved in this model is that mesenchymal cell differentiation into chondrocytes is enhanced in the absense of ASK1, resulting in increased cartilage formation and increased bone formation by endochondral ossification [10]. In the current study, we observed a pronounced enhancement of auricular cartilage regeneration in the ASK1 KO mouse after ear punch. This amount of cartilage regeneration appeared to be significantly greater than what has been reported in other ear regeneration studies [1–3]. In these studies, histology showed newly reformed ear tissue with relatively small cartilage nuggets; while we observed greater than 60% restoration of the cartilage by day 32 in the majority of ears.

Although this report has mainly focused on keratinocytes, other cell types within the ear of the ASK1 KO mouse could also contribute to the observed differences in the wound epidermis, blastemal formation and enhanced cartilage regeneration. Multiple reports in other cells and tissues have detailed how the loss of ASK1 decreases cellular apoptosis, the infiltration of immune cells and pro-inflammatory cytokine production. In contrast, increases in inflammatory cell numbers have been attributed to the enhanced regeneration observed in MRL mice [33], Therefore, more work is necessary to determine the extent to which the immune system is involved in tissue regeneration. It is likely additional factors are involved in conferring regenerative ability in mammals.

## Conclusion

Elucidating the mechanism(s) underlying mammalian regeneration remains an important goal, and although studies in lower vertebrates have yielded valuable insights, a complete understanding requires further study. Based on lower vertebrate studies and our recent findings, we propose that keratinocytes activation may influence the regenerative ability of the wound epidermis and play a critical role in restoring tissue regeneration in mammals. Understanding how the wound epidermis in mammalian regeneration controls blastemal formation and cartilage regeneration in restoring tissue structure and function is of tremendous value for the development of new tissue regeneration strategies.

## Materials and methods

### Mice, ear punch and inhibitor application

ASK1 KO mice were purchased from the Oriental Yeast Co., Tokyo, JP with the permission of Dr. Hidenori Ichijo. The ASK1 KO mice were back-crossed for 10 generations with C57BL/6N (Charles River Laboratories, Frederick, MD). A 2-mm through-and-through hole was made in the center of the cartilaginous part of the ear of ASK1 KO, HET or WT mice using a biopsy punch. The following inhibitors were used at 100uM concentrations in DMSO: ASK1 (NQD1, Sigma, SM0185), p38 (SB203580, Cell Signaling), JNK (SP600125, Sigma, S5567), CAMKII inhibitor (KN93, Calbiochem, 422712). These concentrations were determined through literature search and trial and error approach. Each inhibitor solution or DMSO alone (150ul) was added to the ear punch area in small drops and gently rubbed into both sides of the ear punch with gloved fingers. Inhibitors were applied daily except on weekends for the trial and allowed to dry after application. All experiments were performed under the Institutional Animal Care and Use Committee (IACUC) guidelines and with the approval of the Thomas Jefferson University IACUC committee.

### Western analysis

Ear tissue was lysed in Mammalian Protein Extraction Reagent (MPER, Thermo Fisher, Waltham, MA), and protein concentrations were measured using Bio-Rad Protein Assay (Bio-Rad Laboratories Inc.). Approximately 20 μg of protein were loaded onto an sodium dodecyl sulfate (SDS) polyacrylamide gel and then transferred to a polyvinylidene difluoride (PVDF) membrane. The membrane was blocked in Tris Buffered Saline (TBS) with 0.05% Tween 20 (Thermo Fisher, Waltham, MA) and 5% Membrane Blocking Agent (GE Healthcare, Buckinghamshire, UK) for 1 hour while shaking. Overnight incubation at 4°C was performed with antibodies for either mouse anti-β-actin (Santa Cruz Biotechnology, Dallas, TX) or rabbit anti-ASK1 (Cell Signaling, Danvers, MA) in TBS with 0.05% Tween 20. The primary antibody was removed and the blots were washed three times in TBS with 0.05% Tween 20. Then their respective horseradish peroxidase (HRP) conjugated secondary antibodies (Santa Cruz) were applied to the blots for 1 h at room temperature, washed intensively in TBS with 0.05% Tween 20 and then reacted with ECL Advanced Detection system (Amersham, Pittsburgh, PA) for 5 min at 25°C. Detection of the membranes was done with a FujiFilm Intelligent Darkbox (FujiFilm Co., Tokyo, JP).

### Histology and immunohistochemistry

The ear tissue was excised, fixed overnight with 4% paraformaldehyde (Sigma, St. Louis, MO), and embedded in paraffin wax (Thermo Fisher Scientific Inc., Waltham, MA). Serial 6μm transverse sections from the middle of the wound were stained with hematoxylin and eosin (H&E) and alcian blue (Thermo Fisher Scientific Inc., Waltham, MA) or used for immunohistochemical staining with Diaminobenzidine (DAB). The negative control consisted of a sample incubated with blocking serum and no primary antibody. Deparaffinization, phosphate buffered saline (PBS)washes antigen-retrieval (Vector Laboratories, Burlingame, CA), permeabilization with 0.5% Triton X (Sigma, St. Louis, MO), 3% H_2_O_2_ (Thermo Fisher Scientific Inc., Waltham, MA) in methanol.and PBS wash were applied before blocking in 4% Bovine Serum Albumin (BSA, Equitech-Bio, Kerrville, TX) with 0.1% Tween 20 and incubation with the following primary antibodies made in rabbit; p21 (1:50; Thermo Scientific, Rockford, IL), PCNA (1:100; Bethyl Laboratories, Inc. Montgomery, TX), MMP-13 (1:200; Santa Cruz Biotechnology, Dallas, TX), and filaggrin (PRB-417P, 1:2000, Covance Research Labs, Cumberland, VA) overnight at 4°C. Subsequently, incubation with anti-rabbit secondary antibodies linked to Horseradish Peroxidase (Vector Laboratories, Burlingame, CA) before reacting with DAB (Vector Laboratories, Burlingame, CA) and countrstaining with 50% hematoxylin. Fluorescence immunohistochemistry for was used to localize H2AX (1:50, Bethyl Laboratories, Inc. Montgomery, TX) and cytokeratin 6 (1:100, Novocastra, Newcastle upon Tyne, UK) following the same protocol with an Alexa-fluor 488-conjugated donkey anti-rabbit secondary antibody (Invitrogen, Carlsbad, CA) and mounted with Vectashield Hard Set w/DAPI (Vector Laboratories, Burlingame, CA).

### Image collection and analysis

The ear hole was digitally photographed in a magnetic ear clip (Fig 1B) scale bar every 3–4 days for 32 days. Image analysis provided the area and diameter of the open hole and percentage opening compared to D0 was calculated [34, 35]. Microscopic imaging on a Nikon E800 microscope system (Nikon, Melville, NY) with a 12-bit cooled digital camera (Retiga Exi, QImaging, Burnaby, BC) with an LCD filter to acquire color images. All images were enhanced equally and intensity and localization analysis was performed with a custom written module to automate and standardize the procedure. The percentage length of regenerated cartilage in the newly formed tissue was determined by dividing the length of cartilage by the length of new tissue. All analysis was performed with Image Pro Plus 7.0 (MediaCybernetics, Silver Spring, MD). MD). For all histology analyses 3–5 tissue sections were imaged and analyzed from at least 3 mice/genotype.

### Statistical analysis

A one-way ANOVA (Analysis Of Variance) with post-hoc Tukey HSD (Honestly Significant Difference) Test was used to determine significance between the 3 groups (p values < 0.05) with a 95% confidence interval (http://astatsa.com/OneWay_Anova_with_TukeyHSD/). The data are presented as mean ± SEM. The number of samples used for histology and punch analysis varied from per test and is noted for each experiment, which were performed on both littermates and mice from litters of the same age. Statistical significance is displayed as p < 0.05 (one asterisk), p < 0.01 (two asterisks), p < 0.001 (three asterisks) unless specified otherwise. Brackets indicate the all measurements within are at the significance indicated.

## Supporting information

S1 Fig Filagrin staining in uninjured skin. Staining (dark brown) for the terminal differentiation marker, filaggrin shows no differences in the amount or localization in the uninjured skin from WT and KO mice ears.
(DOC)

## References

[1] Bedelbaeva K, Snyder A, Gourevitch D, Clark L, Zhang XM, Leferovich J, et al Lack of p21 expression links cell cycle control and appendage regeneration in mice. Proc Natl Acad Sci U S A. 2010;107(13):5845–50. Epub 2010/03/17. doi: 10.1073/pnas.1000830107 20231440 10.1073/pnas.1000830107 PMC2851923

[2] Clark LD, Clark RK, Heber-Katz E. A new murine model for mammalian wound repair and regeneration. Clin Immunol Immunopathol. 1998;88(1):35–45. Epub 1998/07/31. S0090122998945196 [pii]. 9683548 10.1006/clin.1998.4519

[3] Shyh-Chang N, Zhu H, Yvanka de Soysa T, Shinoda G, Seligson MT, Tsanov KM, et al Lin28 enhances tissue repair by reprogramming cellular metabolism. Cell. 2013;155(4):778–92. Epub 2013/11/12. doi: 10.1016/j.cell.2013.09.059 [pii]. 24209617 10.1016/j.cell.2013.09.059 PMC3917449

[4] Tamura K, Ohgo S, Yokoyama H. Limb blastema cell: a stem cell for morphological regeneration. Development, growth & differentiation. 2010;52(1):89–99. doi: 10.1111/j.1440-169X.2009.01144.x 19891640 10.1111/j.1440-169X.2009.01144.x

[5] Brockes JP, Kumar A. Appendage regeneration in adult vertebrates and implications for regenerative medicine. Science. 2005;310(5756):1919–23. doi: 10.1126/science.1115200 16373567 10.1126/science.1115200

[6] Cheng CH, Leferovich J, Zhang XM, Bedelbaeva K, Gourevitch D, Hatcher CJ, et al Keratin gene expression profiles after digit amputation in C57BL/6 vs. regenerative MRL mice imply an early regenerative keratinocyte activated-like state. Physiological genomics. 2013;45(11):409–21. doi: 10.1152/physiolgenomics.00142.2012 23512742 10.1152/physiolgenomics.00142.2012 PMC3680783

[7] Fitzgerald J, Rich C, Burkhardt D, Allen J, Herzka AS, Little CB. Evidence for articular cartilage regeneration in MRL/MpJ mice. Osteoarthritis Cartilage. 2008;16(11):1319–26. Epub 2008/05/06. doi: 10.1016/j.joca.2008.03.014 18455447 10.1016/j.joca.2008.03.014

[8] Gourevitch DL, Clark L, Bedelbaeva K, Leferovich J, Heber-Katz E. Dynamic changes after murine digit amputation: the MRL mouse digit shows waves of tissue remodeling, growth, and apoptosis. Wound Repair Regen. 2009;17(3):447–55. Epub 2009/08/08. doi: 10.1111/j.1524-475X.2009.00492.x [pii]. 19660054 10.1111/j.1524-475X.2009.00492.x PMC3645464

[9] Zhang Y, Strehin I, Bedelbaeva K, Gourevitch D, Clark L, Leferovich J, et al Drug-induced regeneration in adult mice. Sci Transl Med. 2015;7(290):290ra92 doi: 10.1126/scitranslmed.3010228 26041709 10.1126/scitranslmed.3010228 PMC4687906

[10] Eaton GJ, Zhang QS, Diallo C, Matsuzawa A, Ichijo H, Steinbeck MJ, et al Inhibition of apoptosis signal-regulating kinase 1 enhances endochondral bone formation by increasing chondrocyte survival. Cell death & disease. 2014;5:e1522 doi: 10.1038/cddis.2014.480 25393478 10.1038/cddis.2014.480 PMC4260738

[11] Zhang QS, Eaton GJ, Diallo C, Freeman TA. Stress-Induced Activation of Apoptosis Signal-Regulating Kinase 1 Promotes Osteoarthritis. J Cell Physiol. 2015 doi: 10.1002/jcp.25186 26405834 10.1002/jcp.25186 PMC5952048

[12] Harada C, Nakamura K, Namekata K, Okumura A, Mitamura Y, Iizuka Y, et al Role of apoptosis signal-regulating kinase 1 in stress-induced neural cell apoptosis in vivo. Am J Pathol. 2006;168(1):261–9. Epub 2006/01/10. S0002-9440(10)62088-3 [pii] doi: 10.2353/ajpath.2006.050765 16400028 10.2353/ajpath.2006.050765 PMC1592659

[13] Ichijo H, Nishida E, Irie K, ten Dijke P, Saitoh M, Moriguchi T, et al Induction of apoptosis by ASK1, a mammalian MAPKKK that activates SAPK/JNK and p38 signaling pathways. Science. 1997;275(5296):90–4. Epub 1997/01/03. 8974401 10.1126/science.275.5296.90

[14] Kawarazaki Y, Ichijo H, Naguro I. Apoptosis signal-regulating kinase 1 as a therapeutic target. Expert Opin Ther Targets. 2014;18(6):651–64. Epub 2014/03/26. doi: 10.1517/14728222.2014.896903 24660755 10.1517/14728222.2014.896903

[15] Sayama K, Hanakawa Y, Shirakata Y, Yamasaki K, Sawada Y, Sun L, et al Apoptosis signal-regulating kinase 1 (ASK1) is an intracellular inducer of keratinocyte differentiation. J Biol Chem. 2001;276(2):999–1004. doi: 10.1074/jbc.M003425200 11029458 10.1074/jbc.M003425200

[16] Gourevitch D, Clark L, Chen P, Seitz A, Samulewicz SJ, Heber-Katz E. Matrix metalloproteinase activity correlates with blastema formation in the regenerating MRL mouse ear hole model. Dev Dyn. 2003;226(2):377–87. Epub 2003/01/31. doi: 10.1002/dvdy.10243 12557216 10.1002/dvdy.10243

[17] Freedberg IM, Tomic-Canic M, Komine M, Blumenberg M. Keratins and the keratinocyte activation cycle. J Invest Dermatol. 2001;116(5):633–40. doi: 10.1046/j.0022-202x.2001.doc.x 11348449 10.1046/j.1523-1747.2001.01327.x

[18] Sayama K, Komatsuzawa H, Yamasaki K, Shirakata Y, Hanakawa Y, Ouhara K, et al New mechanisms of skin innate immunity: ASK1-mediated keratinocyte differentiation regulates the expression of beta-defensins, LL37, and TLR2. Eur J Immunol. 2005;35(6):1886–95. doi: 10.1002/eji.200425827 15864780 10.1002/eji.200425827

[19] Shyh-Chang N, Daley GQ. Lin28: primal regulator of growth and metabolism in stem cells. Cell Stem Cell. 2013;12(4):395–406. Epub 2013/04/09. doi: 10.1016/j.stem.2013.03.005 [pii]. 23561442 10.1016/j.stem.2013.03.005 PMC3652335

[20] Chew YC, Adhikary G, Wilson GM, Reece EA, Eckert RL. Protein kinase C (PKC) delta suppresses keratinocyte proliferation by increasing p21(Cip1) level by a KLF4 transcription factor-dependent mechanism. J Biol Chem. 2011;286(33):28772–82. doi: 10.1074/jbc.M110.205245 21652709 10.1074/jbc.M110.205245 PMC3190685

[21] Wong PP, Pickard A, McCance DJ. p300 alters keratinocyte cell growth and differentiation through regulation of p21(Waf1/CIP1). PLoS One. 2010;5(1):e8369 doi: 10.1371/journal.pone.0008369 20084294 10.1371/journal.pone.0008369 PMC2805707

[22] Carlo MD Jr., Loeser RF. Increased oxidative stress with aging reduces chondrocyte survival: correlation with intracellular glutathione levels. Arthritis Rheum. 2003;48(12):3419–30. doi: 10.1002/art.11338 14673993 10.1002/art.11338

[23] Santini MP, Talora C, Seki T, Bolgan L, Dotto GP. Cross talk among calcineurin, Sp1/Sp3, and NFAT in control of p21(WAF1/CIP1) expression in keratinocyte differentiation. Proc Natl Acad Sci U S A. 2001;98(17):9575–80. doi: 10.1073/pnas.161299698 11493684 10.1073/pnas.161299698 PMC55494

[24] Stuart RO, Sun A, Bush KT, Nigam SK. Dependence of epithelial intercellular junction biogenesis on thapsigargin-sensitive intracellular calcium stores. J Biol Chem. 1996;271(23):13636–41. 8662885 10.1074/jbc.271.23.13636

[25] Nigam SK, Rodriguez-Boulan E, Silver RB. Changes in intracellular calcium during the development of epithelial polarity and junctions. Proc Natl Acad Sci U S A. 1992;89(13):6162–6. 1631104 10.1073/pnas.89.13.6162 PMC402142

[26] Denning MF, Dlugosz AA, Williams EK, Szallasi Z, Blumberg PM, Yuspa SH. Specific protein kinase C isozymes mediate the induction of keratinocyte differentiation markers by calcium. Cell Growth Differ. 1995;6(2):149–57. 7756173

[27] Bikle DD, Xie Z, Tu CL. Calcium regulation of keratinocyte differentiation. Expert Rev Endocrinol Metab. 2012;7(4):461–72. doi: 10.1586/eem.12.34 23144648 10.1586/eem.12.34 PMC3491811

[28] Takahashi H, Asano K, Manabe A, Kinouchi M, Ishida-Yamamoto A, Iizuka H. The alpha and eta isoforms of protein kinase C stimulate transcription of human involucrin gene. J Invest Dermatol. 1998;110(3):218–23. doi: 10.1046/j.1523-1747.1998.00110.x 9506439 10.1046/j.1523-1747.1998.00110.x

[29] Efimova T, Deucher A, Kuroki T, Ohba M, Eckert RL. Novel protein kinase C isoforms regulate human keratinocyte differentiation by activating a p38 delta mitogen-activated protein kinase cascade that targets CCAAT/enhancer-binding protein alpha. J Biol Chem. 2002;277(35):31753–60. doi: 10.1074/jbc.M205098200 12080077 10.1074/jbc.M205098200

[30] Schumacher M, Schuster C, Rogon ZM, Bauer T, Caushaj N, Baars S, et al Efficient keratinocyte differentiation strictly depends on JNK-induced soluble factors in fibroblasts. J Invest Dermatol. 2014;134(5):1332–41. doi: 10.1038/jid.2013.535 24335928 10.1038/jid.2013.535

[31] Li W, Nadelman C, Henry G, Fan J, Muellenhoff M, Medina E, et al The p38-MAPK/SAPK pathway is required for human keratinocyte migration on dermal collagen. J Invest Dermatol. 2001;117(6):1601–11. doi: 10.1046/j.0022-202x.2001.01608.x 11886529 10.1046/j.0022-202x.2001.01608.x

[32] Stoll SW, Kansra S, Elder JT. Keratinocyte outgrowth from human skin explant cultures is dependent upon p38 signaling. Wound Repair Regen. 2003;11(5):346–53. 12950638 10.1046/j.1524-475x.2003.11506.x

[33] Heber-Katz E, Gourevitch D. The relationship between inflammation and regeneration in the MRL mouse: potential relevance for putative human regenerative(scarless wound healing) capacities? Ann N Y Acad Sci. 2009;1172:110–4. Epub 2009/09/09. doi: 10.1111/j.1749-6632.2009.04499.x [pii]. 19735244 10.1111/j.1749-6632.2009.04499.x PMC3646560

[34] Deoliveira D, Jiao Y, Ross JR, Corbin K, Xiao Q, Toncheva G, et al An ear punch model for studying the effect of radiation on wound healing. Int J Radiat Biol. 2011;87(8):869–77. Epub 2011/04/13. doi: 10.3109/09553002.2011.568575 21480768 10.3109/09553002.2011.568575 PMC3193941

[35] Kench JA, Russell DM, Fadok VA, Young SK, Worthen GS, Jones-Carson J, et al Aberrant wound healing and TGF-beta production in the autoimmune-prone MRL/+ mouse. Clin Immunol. 1999;92(3):300–10. Epub 1999/09/10. doi: 10.1006/clim.1999.4754 [pii]. 10479535 10.1006/clim.1999.4754

