# Supplementary material for: Inhibition of apoptosis signal-regulating kinase 1 alters the wound epidermis and enhances auricular cartilage regeneration
Source: PLoS One. 2017 Oct 18;12(10):e0185803. doi: 10.1371/journal.pone.0185803 (PMC5646791; doi:10.1371/journal.pone.0185803)
Supplement: S1 Fig — Staining (dark brown) for the terminal differentiation marker, filaggrin shows no differences in the amount or localization in the uninjured skin from WT and KO mice ears. (DOC) [file pone.0185803.s001.doc]

**Inhibition of Apoptosis Signal-regulating Kinase 1 alters the Wound Epithelium and Enhances Auricular Cartilage Regeneration**

Qian-Shi Zhang, Deepa S Kurpad, My G Mahoney, Marla J Steinbeck, Theresa A Freeman


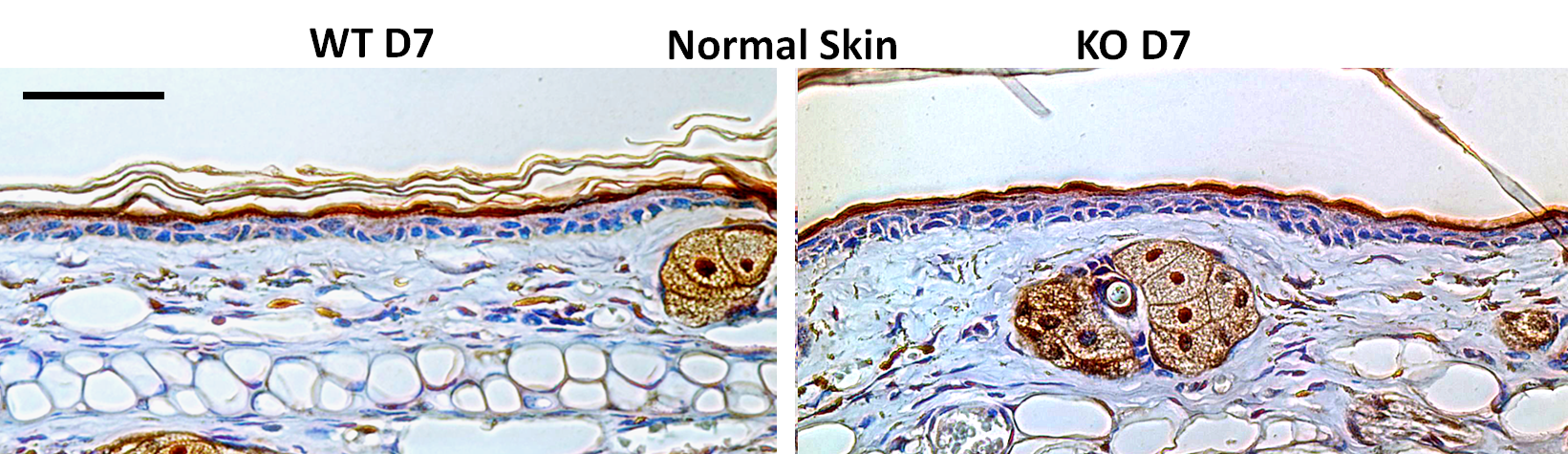


**Filagrin Staining in uninjured skin.** Staining (dark brown) for the terminal differentiation marker, filaggrin shows no differences in the amount or localization in the uninjured skin from WT and KO mice ears.
